# An online survey of primary care physicians’ knowledge of common respiratory diseases in China

**DOI:** 10.1038/s41533-022-00289-5

**Published:** 2022-08-19

**Authors:** Zihan Pan, Ting Yang, Chunhua Chi, Yahong Chen, Jiping Liao, Kewu Huang, Siân Williams, Chen Wang

**Affiliations:** 1 Department of Pulmonary and Critical Care Medicine, Peking University Third Hospital, Beijing, People’s Republic of China; 2 General Practice, Peking University First Hospital, Beijing, People’s Republic of China; 3 Research Center for Chronic Airway Diseases, Peking University Health Science Center, Beijing, People’s Republic of China; 4 Department of Pulmonary and Critical Care Medicine, China-Japan Friendship Hospital, Beijing, People’s Republic of China; 5 National Clinical Research Center for Respiratory Diseases, Beijing, People’s Republic of China; 6 Institute of Respiratory Medicine, Beijing, People’s Republic of China; 7 Chinese Academy of Medical Science, Beijing, People’s Republic of China; 8 Department of Pulmonary and Critical Care Medicine, Peking University First Hospital, Beijing, People’s Republic of China; 9 International Primary Care Respiratory Group, London, UK; 10 Chinese Academy of Medical Sciences & Peking Union Medical College, Beijing, People’s Republic of China

**Keywords:** Chronic obstructive pulmonary disease, Epidemiology

## Abstract

China has a huge population with respiratory diseases, these diseases should be managed well in primary care, however, primary care physicians’ knowledge level of these diseases were unknown. The aim of the study was to assess primary care physicians’ knowledge of asthma, CAP, COPD, and influenza in China. An e-questionnaire was distributed to attendees of respiratory diseases academic conferences in China from July, 2017 to December, 2018. 7391 questionnaires were returned and 4815 valid questionnaires were analyzed, 3802 (79.0%) from community health service centers and 1013 (21.0%) from township hospitals. The average score of the questionnaire was 83.3 (±20.397) and 72.1 (±20.898) in township and community hospitals, respectively (*P* < 0.05). 61.4%, 48.7%, and 42.5% of the primary care physicians were aware of clinical manifestations of COPD, asthma, and simple influenza. 85.7%, 8.1%, 16.1%, and 1.0% knew how to diagnose COPD, asthma, CAP and influenza, respectively. 94.4% of the physicians lacked the knowledge of treating COPD with bronchodilators; 53.7% knew non-pharmacological treatments for COPD. 73.6% were unable to deal with asthma attacks. 65.1% did not know what the most essential and important treatment for influenza was. 92% of physicians did not know the management for stable COPD; 3.0% knew all prevention and management measures for asthma. 37.9% knew all the preventive measures for CAP. 44.9% did not know the important role of influenza vaccine in preventing influenza and its complications. Primary care physicians in China had a poor knowledge of CAP, asthma, Influenza, COPD. There is a need for improved training of common respiratory diseases.

## Introduction

Respiratory diseases are the most common diseases in China^1^ and large amounts of medical and societal resources have been spent on these diseases. On the one hand, there is a tremendous population with chronic non-communicable respiratory diseases (CRD). It is estimated that there are 99 million patients with chronic obstructive pulmonary disease (COPD)^2^ and 43.7 million patients with asthma in China^3^, with a prevalence of 13.7%^2^ and 4.3%^3^, respectively. On the other hand, the incidence and mortality of acute communicable respiratory diseases are also rising yearly in China. The incidence of influenza has increased from 14.9/100,000 in 2009 to 33.1/100,000 in 2017; the excess mortality of all causes caused by influenza was (6.9–17.2)/100,000^4^. What’s more, influenza virus ranks first among viruses causing community-acquired pneumonia (CAP)^5^. In recent years, China has even experienced several outbreaks of influenza and the increasing excess mortality of influenza has caused a kind of social panic^6,7^. However, up to 2017, there were only <8000 respiratory physicians registered in China^1^. The outbreak of coronavirus disease 2019 (COVID-19) has once again made us aware of the importance of primary care, and let us begin to think about the role of primary care in dealing with infectious respiratory diseases. COVID-19 pandemic also posted a huge challenge to the health system. It is far from enough for these respiratory physicians to manage more than 140 million people with common respiratory diseases in China, let alone in COVID-19 context. Even though the primary care in China played an important role and made great contribution in fighting against COVID-19^8^, it was also with core responsibilities in preventing and managing common diseases.

Primary care is the cornerstone of a health system. The two important representatives of primary care in China are township hospitals and community health service centers^9^. In recent years, national strategies have been released to promote and enhance the development of primary care in China^10,11^. Meanwhile, guidelines for the management of CAP^5,12^ and influenza^13^ in primary care are updated as conditions change. Furthermore, chronic respiratory diseases’ prevention and treatment is one of the major actions of Healthy China 2030 Action Plan^14^. However, there were gaps in the quality of primary care in China, insufficient training and educational opportunities for the practitioners were one of the deficiencies, and tailoring continuing training for primary care workforce was considered as one of the top priorities to deal with the challengeable situation^15^. Obviously, knowledge assessment was the first step before any effective intervention was conducted, and to improve the management quality of common respiratory diseases in primary care, the current knowledge level of primary care physicians should be assessed. The Chinese Alliance for Respiratory Diseases in Primary Care (CARDPC) is the leading academic organization in respiratory diseases in primary care. And it has undertaken a series of efforts to improve the ability of primary care physicians to manage common respiratory diseases, including the national touring training and education project-*Action Now*, which referred to CAP, COPD, Asthma, Influenza Knowledge Assessment among primary care physicians in China^16^. The paper was to report the baseline results of the project.

An online questionnaire survey was conducted from 2017 to 2018 aiming at comprehensively exploring the knowledge level of primary care physicians in China on the four common respiratory diseases. We aimed to identify primary care physicians’ knowledge weakness and use the findings to design further vocational training programs.

## Results

It was roughly estimated that between 2017 and 2018, more than 10,000 people participated in various academic activities organized by CARDPC in nearly 30 provinces across China. 7391 attendees returned the questionnaire, 5405 (73.1%) questionnaires were returned in 2017 and 1986 (26.9%) in 2018. 7353 (99.5%) questionnaires were returned via Wechat and 38 (0.5%) via web link. Since it was not possible to calculate the total visits to the questionnaire, the specific response rate was unclear. Responses were eligible if the respondent was:

- physician working in community health service centers or township hospitals (including affiliated satellite sites), like General Practitioner (GP), respiratory physician, Traditional Chinese–West medical physician, and internal physician;
- managing respiratory diseases in community health service centers or township hospitals (including affiliated satellite sites); and
- completely completed the questionnaire.

**Fig. 1:**
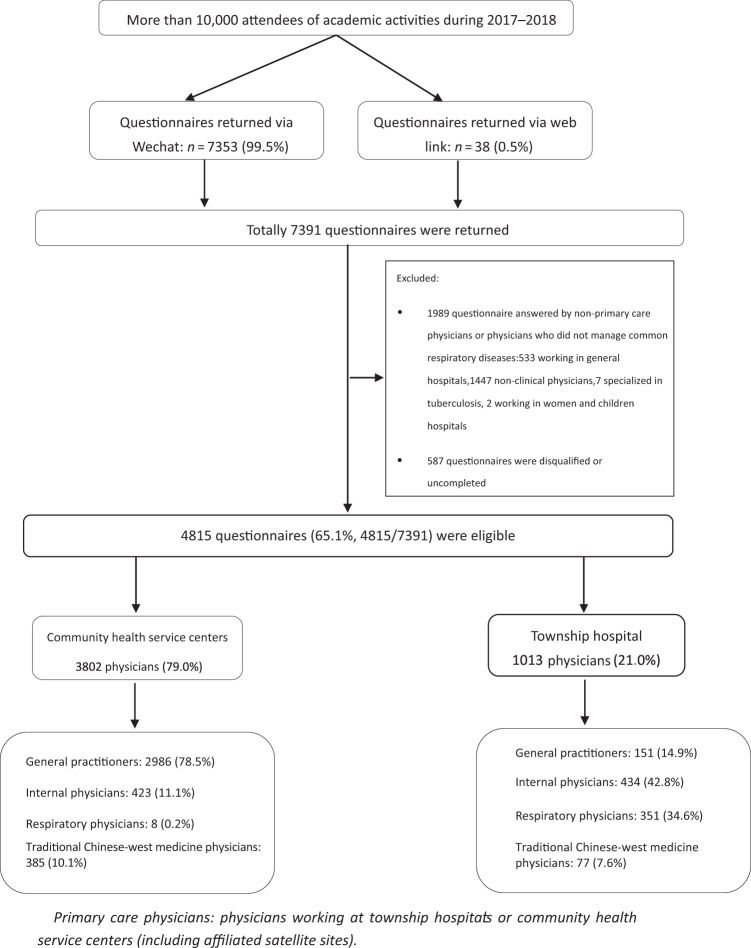
Flow chart of respondents.

26.9% (*n* = 1989) of the questionnaires answered by non-primary care physicians or physicians who did not manage common respiratory diseases, 7.9% (*n* = 587) were disqualified or uncompleted with missing data. This resulted in 4815 (65.1%) eligible responses (Fig. 1).

### Characteristics of respondents

Overall, 64.6% (*n* = 3112) of respondents were women, the mean age of the sample was 38.7 years old. 92.1% (*n* = 4435) of respondents were younger than 50 years old. 24.4% (*n* = 1174) of them were not qualified to Bachelor’s degree with a mean age of 42.9 years old, amongst whom 90% (*n* = 1057) finished junior college which referred to a 3-year vocational training program. 67.0% (*n* = 3228) of them had a Bachelor degree who had completed a systematic 5-year medical education program, 8.6% (*n* = 413) had a master degree who had completed an extra 3-year national advanced vocational training program after the 5-year medical education program. 38.8% (*n* = 1869) respondents had been in practice for <10 years, 47.9% (*n* = 2307) had an intermediate vocational title, 65.2% (*n* = 3138) of them were specialized in General Practice. 71.1% (*n* = 3802) of respondents practiced in community health service centers (including the affiliated satellite sites). Majority (*n* = 3384, 70.3%) of the respondents were from north of China, only 0.3% (*n* = 15) were from south of China (Table 1).

**Table 1:** Demographic characteristics of respondents.

Item	Primary care physicians 4815 (100.0)				Non-primary care physicians or physicians who did not manage common respiratory diseases *n* (%) 1989 (100.0)
	Community health service centers *n* (%) 3802 (71.1)	Township hospital *n* (%) 1013 (18.9)	Total number *n* (%) 4815 (100.0)	*P*	
*Gender*					
Male *n* (%)	1225 (32.2)	478 (47.2)	1703 (35.4)	0.000	541 (27.2)
Female *n* (%)	2577 (67.8)	535 (52.8)	3112 (64.6)		1448 (72.8)
*Age*					
Mean age (years)	39.4	36.0	38.7	–	34.2
20–30 *n* (%)	570 (15.0)	286 (28.2)	856 (17.8)	0.000	732
31–39 *n* (%)	1401 (36.8)	419 (41.4)	1820 (37.8)		603
40–49 *n* (%)	1524 (40.1)	235 (23.2)	1759 (36.5)		513
≥50 *n* (%)	301 (7.9)	71 (7.0)	372 (7.7)		137
Unavailable data (*n*)	6	2	8 (0.2)	–	4
*Education level*					
High school or below *n* (%)	110 (2.9)	7 (0.7)	117 (2.4)	0.000	49 (2.5)
Junior college *n* (%)	952 (25.0)	105 (10.4)	1057 (22.0)		537 (27.0)
Bachelor *n* (%)	2429 (63.9)	799 (78.9)	3228 (67.0)		1111 (55.9)
Master *n* (%)	311 (8.2)	102 (10.1)	413 (8.6)		292 (14.7)
*Experience years*					
≤10 *n* (%)	1321 (34.7)	548 (54.1)	1869 (38.8)	0.000	1042 (52.4)
10–20 *n* (%)	1193 (31.4)	281 (27.2)	1474 (30.6)		463 (23.2)
20–30 *n* (%)	1105 (29.1)	154 (15.2)	1259 (26.1)		397 (20.0)
>30 *n* (%)	183 (4.8)	31 (3.1)	214 (4.4)		87 (4.4)
*Vocational title*					
No *n* (%)	111 (2.9)	88 (8.7)	199 (4.1)	0.000	293 (14.7)
Junior *n* (%)	1208 (31.8)	304 (30.0)	1512 (31.4)		719 (36.1)
Intermediate *n* (%)	1889 (49.7)	418 (41.3)	2307 (47.9)		667 (33.5)
Senior *n* (%)	594 (15.6)	203 (20.0)	797 (16.6)		310 (15.6)
*Specialty*					
General practitioner *n* (%)	2987 (78.6)	151 (14.9)	3138 (65.2)	0.000	Working in general hospitals *n* = 533 (26.8)
Traditional Chinese and west medicine *n* (%)	384 (10.1)	77 (7.6)	461 (9.6)		Non-clinical physicians *n* = 1447 (72.8)
Respiratory physician *n* (%)	8 (0.2)	351 (34.6)	359 (7.5)		Specialized in tuberculosis *n* = 7 (0.4)
Internal physician *n* (%)	423 (11.1)	434 (42.8)	857 (17.8)		Working in women and children hospitals *n* = 2 (0.1)
*Location of current practice*					
Northeast *n* (%)	49 (1.3)	69 (6.8)	118 (2.5)	0.000	152 (7.6)
East *n* (%)	381 (10.0)	277 (27.3)	658 (13.7)		367 (18.5)
North *n* (%)	3168 (83.3)	216 (21.3)	3384 (70.3)		763 (38.4)
Central China *n* (%)	47 (1.2)	159 (15.7)	206 (4.3)		186 (9.4)
South *n* (%)	3 (0.1)	12 (1.2)	15 (0.3)		5 (0.3)
Southwest *n* (%)	67 (1.8)	165 (16.3)	232 (4.8)		359 (18.0)
Northwest *n* (%)	87 (2.3)	115 (11.4)	202 (4.2)		157 (7.9)

### Knowledge evaluation

Overall, the average score of all the 4815 respondents was 74.5 (±21.287), 39.2% (74.47/190) of the total score of the questionnaire, 83.3 (±20.397) (43.8%, 83.3/190), and 72.1 (±20.898) (38.0%, 72.1/190) in township and community hospitals (*P* < 0.05), respectively. The best answered section was COPD, with an average score of 53.4% (32.0/60) of its section, the average score of asthma, CAP, and influenza were only 38.2% (17.2/45), 26.1% (10.4/40), and 32.9% (14.8/45) of the relative parts, respectively. Physicians in township hospitals had higher scores. Scores of diseases are shown in Table 2.

**Table 2:** Scores of four diseases among physicians.

Institutions	Total (*N*= 4815)		Community health service centers (*N* = 3802)		Township hospital (*N* = 1013)	
Items	Mean score (±SD)	95% CI	Mean score (±SD)	95% CI	Mean score (±SD)	95% CI
Total (190 points)	74.5 (±21.287)	(73.87,75.07)	72.1 (±20.898)	(71.45,72.78)	83.3 (±20.397)	(82.05,84.57)
COPD^a^ (60 points)	32.0 (±9.855)	(32.29,32.83)	30.8 (±9.49)	(30.50,31.10)	36.7 (±9.786)	(36.08,37.29)
Asthma^a^ (45 points)	17.2 (±7.459)	(17.24,17.64)	16.4 (±7.315)	(16.20,16.66)	20.0 (±7.317)	(19.55,20.46)
CAP^a^ (40 points)	10.4 (±6.741)	(10.35,10.71)	10.1 (±6.822)	(9.85,10.29)	11.8 (±6.249)	(11.41,12.18)
Influenza^b^ (45 points)	14.8 (±7.072)	(14.57,14.95)	14.8 (±7.103)	(14.58,15.04)	14.8 (±6.960)	(14.40,15.26)

^a^Denote significant difference between community and township hospitals, *p* = 0.000.

^b^Denote no significant difference between community and township hospitals, *p* = 0.192.

61.4%, 48.7%, and 42.5% of respondents were aware of the characteristic symptoms or clinical manifestations of COPD, asthma, and simple influenza, respectively. Meanwhile, 49.2% of them knew the most important cause of COPD, 22.0% of them knew the common pathogens of CAP, and 16.4% knew the epidemiological characteristics of influenza.

85.7% of the respondents were aware of the diagnosis standard of COPD, however, 45.3% of them did not know the criteria of GOLD II COPD. 8.1% of them were aware of how to diagnose asthma, 16.1% were able to correctly make the clinical diagnosis of CAP. (2.1%, 100/4815), (1.0%, 46/4815), and (0.5%, 25/4815) of the respondents were aware of the pathogenic diagnostic specimens of CAP, the standardized diagnosis of influenza, and the diagnostic criteria for severe influenza.

94.4% (*n* = 4546) of the respondents did not choose bronchodilators for COPD correctly; 53.7% (*n* = 2587) of them knew the non-pharmacological treatments for COPD. Furthermore, 73.6% (*n* = 3543) of respondents were unable to properly deal with asthma attacks; only 9.4% (*n* = 453) of them chosen the right treatment plan for newly diagnosed mild asthma patients. For young adults with no underlying disease, 2.1% (*n* = 102) of respondents correctly selected all of the initial empirical anti-infective therapy for CAP. 34.9% (*n* = 1680) knew what the most essential and important treatment for influenza is.

92% (*n* = 4429) did not pick out all the management for stable COPD; 27.7% of them knew the duration of daily oxygen inhalation (>15 h) for long-term home oxygen therapy. Only 3.0% (*n* = 144) of respondents selected all prevention and management measures for asthma. 29.3% (*n* = 1410) knew all the preventive measures for CAP. For influenza, 44.9% (*n* = 2161) did not know the important role of influenza vaccine in preventing influenza and its complications.

Correct rates of each item in each disease were shown in Table 3 (COPD and asthma) and Table 4 (CAP and influenza).

**Table 3:** Correct rates of items of COPD and asthma.

Item	Total		Community health service centers		Township hospital	
	*N*	%	*N*	%	*N*	%
Q11 Characteristic symptoms of COPD^a^	2968	61.4	2189	57.6	779	76.9
Q12 The most important environmental factors of COPD^a^	2367	49.2	1771	46.6	596	58.8
Q13 Diagnosis standard of COPD^a^	2793	58.0	2035	53.5	758	74.8
Q14 The gold standard tool for diagnosing COPD^a^	4125	85.7	3185	83.8	940	92.8
Q15 The standard of Grade II COPD^a^	2632	54.7	1930	50.8	702	69.3
Q16 The Main treatment to control the symptoms of COPD^a^	3968	82.4	3119	82.0	849	83.8
Q17 The main types of bronchodilators for the treatment of COPD^a^	269	5.6	193	5.1	76	7.5
Q18 The daily duration of long-term family oxygen therapy^a^	1334	27.7	857	22.5	477	47.1
Q19 The management objectives of COPD in stable phase^a^	3417	71.0	2758	72.5	659	65.1
Q20 Management of COPD in stable phase^a^	386	8.0	316	8.3	70	6.9
Q21 The most common cause of acute exacerbation of COPD^a^	4009	83.3	3136	82.5	873	86.2
Q22 The non-pharmacological treatment measures for COPD^a^	2587	53.7	1934	50.9	653	64.5
Q23 The main clinical manifestations of asthma^a^	2343	48.7	1754	46.1	589	58.1
Q24 The diagnosis of asthma^a^	3932	81.7	3055	80.4	877	86.6
Q25 Auxiliary examinations of asthma^a^	391	8.1	288	7.6	103	10.2
Q26 The normal value of peak flow meter evaluation^a^	2521	52.4	1840	48.4	681	67.2
Q27 The first choice of medication of asthma control^a^	3097	64.3	2323	61.1	774	76.4
Q28 The remission medications for asthma^a^	2397	49.8	1823	47.9	574	56.7
Q29 The treatment options for patients with mild asthma^a^	453	9.4	351	9.2	102	10.1
Q30 The principles for the treatment of asthmatic attack^a^	1272	26.4	927	24.4	345	34.1
Q31 Prevention and management measures of asthma^a^	144	3.0	123	3.2	21	2.1

^a^Denote significant difference between community and township hospitals, *p* = 0.000.

**Table 4:** Correct rates of items of CAP and influenza.

Item	Total		Community health service centers		Township hospital	
	*N*	%	*N*	%	*N*	%
Q32 The clinical diagnosis basis of community-acquired pneumonia^a^	774	16.1	514	13.5	260	25.7
Q33 Etiological diagnosis specimens of community-acquired pneumonia^a^	100	2.1	69	1.8	31	3.1
Q34 The conditions that CAP patients need etiological examinations^a^	1875	38.9	1446	38.0	429	42.3
Q35 The diagnostic criterion of severe pneumonia^a^	2903	60.3	2157	56.7	746	73.6
Q36 The common pathogens of CAP^a^	1057	22.0	832	21.9	225	22.2
Q37 The preferred anti-infective drug^a^	102	2.1	77	2.0	25	2.5
Q38 The signs of response to treatment^a^	1826	37.9	1477	38.8	349	34.5
Q39 The preventions of pneumonia^a^	1410	29.3	1086	28.6	324	32.0
Q40 Symptoms and signs of simplex influenza^a^	2045	42.5	1644	43.2	401	39.6
Q41 The epidemiological characteristics of influenza^a^	790	16.4	574	15.1	216	21.3
Q42 The main infection routes of influenza^a^	3041	63.2	2413	63.5	628	62.0
Q43 The diagnostic criterion for influenza^a^	46	1.0	9	0.2	37	3.7
Q44 The criterion of severe influenza^a^	25	0.5	25	0.7	0	0.0
Q45 The most common and serious complications of influenza^a^	3276	68.0	2548	67.0	728	71.9
Q46 The most basic and important link of the treatment of influenza^a^	1680	34.9	1225	32.2	455	44.9
Q47 Drugs can be used to treat influenza B^a^	483	10.0	320	8.4	163	16.1
Q48 The most effective way to prevent influenza and its complications^a^	2654	55.1	2211	58.2	443	43.7

^a^Denote significant difference between community and township hospitals, *p* *=* 0.000.

## Discussion

Primary care is the essential element of a health system and it is the first line in managing respiratory diseases. Primary care physicians’ knowledge is an extremely important factor for the goal to improve patients’ prognosis and quality of life. Some sporadic studies have evaluated the knowledge of primary care physicians on COPD and asthma, but no persuasive studies have assessed their knowledge of influenza and CAP. This was the largest national evaluation of its kind which evaluated COPD, asthma, influenza, and CAP simultaneously in China. The results provided a robust reference for the design of a series of systematic training courses on respiratory diseases for physicians in primary care in China or similar settings. Furthermore, this study may also trigger the evaluation of the use and availability of pulmonary rehabilitation, spirometry, inhaler medications, and other treatment devices.

This study represented a first step toward understanding awareness of four most common respiratory diseases among primary care physicians in China, with the underlying objective to identify weak areas in current primary care practice where further educational resources might lead to more targeted training programs and improved patient outcomes. Even though the accurate response rate was unclear, we got an impressive large sample of 7391 returned questionnaires, and 4815 (65.1%) questionnaires were eligible and analyzed.

It was clear from the results of the study that the primary care physicians’ knowledge of CAP, asthma, Influenza, COPD was inadequate. On the whole, both the average score of the whole questionnaire and the average score of each disease was <60% of the corresponding part, suggesting that the respondents did not even master the basic knowledge of these diseases. Foremost, respondents cannot correctly identify the clinical features of the four diseases. Low proportions of them were aware of the clinical manifestations of COPD (61.4%), asthma (48.7%), and influenza (42.5%). Meanwhile, less than half of them knew the cause of COPD, the common pathogens of CAP, and the epidemiological characteristics of influenza. This finding was important in view of the fact that, in primary care settings, these diseases were primarily diagnosed clinically, based on epidemiological characteristics, clinical symptoms, or signs after the exclusion of other respiratory conditions, such as tuberculosis. Although almost half (45.3%) of the respondents did not know the grade criteria for COPD, it was gratifying to see that more than 85% of them knew the diagnostic criteria of COPD in theory, while only 8.1%, 16.1%, and 1.0% of them could make the right diagnosis of asthma, CAP and influenza, respectively. The high correct rate of COPD diagnostic criteria was probably attributed to a series of policies on COPD in recent years^17–20^, including the national policy *The 13th Five-Year Plan for Health Care* which recommended the incorporation of pulmonary function tests into routine health examinations^17^, in 2014 COPD was included in the national chronic disease surveillance system^18^, in 2015 COPD was included in the national work plan for the prevention and treatment of chronic diseases^19^ and in 2016 COPD was included in the hierarchical diagnosis and treatment program^20^. Nevertheless, COPD was the only respiratory disease mentioned in the above national projects, which have increased and strengthened the relative training on COPD, but the results also indicated that further training was still needed as primary care physicians’ knowledge of COPD treatment was not optimistic.

Even though physicians in township hospitals had a better knowledge of these diseases than community health centers, the knowledge level of influenza between them was similar, which may be due to the incidence of influenza has increased yearly in China so that the government attached great importance to influenza training. There is a National Free Influenza Vaccination Program for people over 65 years old, which encourages primary care physicians to exhort patients to be vaccinated against influenza in the flu seasons and also urges them to apply what they have learned repeatedly in clinic. In terms of treatment, the knowledge was even worse. Compared with the high correct rate of COPD diagnosis, a very small proportion of physicians were aware of bronchodilators for COPD. As the most two common chronic non-communicable respiratory diseases, only 8% and 3% were aware of the management of stable COPD and asthma, respectively.

Although primary care physicians had a poor knowledge of the four diseases, 75.6% of the respondents were qualified with a bachelor (67%) or even master (8.6%) degree in medicine, only 2.4% were qualified less than junior degree in total, with 2.9% in community health service centers and 0.7% in township hospitals, meanwhile, the qualification of professionalism was much higher than the national level. The National statistics data showed that there were 25% of primary care physicians in community health centers and 42% of those in township hospitals had less than a junior medical college level of education (junior medical college was lower than Bachelor degree, it was the basic requirement for a licensed assistant physician) in 2018^15^. It indicated that the respondents of the study should be proficient in this knowledge.

What should be mentioned here was that the medical education system was different in China from most western countries, where physicians were required to complete training at universities including completion of master’s degree, while primary care physicians refers to people with not a bachelor’s degree, but having completed a vocational training program. In China, when the person finished the 5-year systematically medical education then he/she was qualified with a bachelor’s degree of medicine. Moreover, completion of the 5-year study in medical college was basically required to become a licensed doctor or physician. They could be qualified with a master degree if they finish a 3-year advanced vocational training program after the 5-year study. However, usually, higher qualification the physicians can obtain, higher medical institutions they will choose. In the study, we can see that more than two-thirds of these physicians with a bachelor or above degree worked in primary care, which was a relatively high proportion in the context of primary care in China. Due to the new round of medical reform in China attached great importance to the development of primary care, physicians of primary care were in a period of replacement between the young and the old generation, but we also hope that more physicians with high qualifications are willing to work in primary care facilities.

General practitioners or primary care physicians are the mainstay in primary care facilities in China, the government has paid great attention to its development. In 2018, the State Office issued the policy on *Reforming and improving the incentive Mechanism for the training and using of General Practitioners*^21^, which put forward the specific goal of the development of General Practitioners or primary care physicians—by 2020, the number of General Practitioners would reach 300,000, and there would be 2 to 3 qualified General Practitioners for every 10,000 residents in urban and rural areas. By 2030, the number of General Practitioners should reach 700,000, and there will be 5 qualified general practitioners for every 10,000 residents in urban and rural areas. To achieve the above goals as scheduled, the state has formulated different post-graduate training programs. The current training model was mainly based on the “5-year college education plus 3-year standardized post-graduate resident training” as the main body, and the “3-year college education plus 2-year assistant general practitioner training” as the supplement for people without bachelor degree. In addition, the state has also expanded the approaches to train General Practitioners, such as the implementation of the special post-graduation program for general practitioners, job transfer training, free training of rural order-oriented medical students, and so on^9^.

In the past few decades, hypertension and diabetes were the two conditions that were heavily prioritized in primary care settings in China, respiratory diseases were in a relatively weak position^15^. That may lead to less opportunities for physicians to apply the relevant knowledge in the clinical practice. Although we did not explore factors affecting their knowledge, we thought that may be one important factor for the poor knowledge of the respondents.

There were studies to investigate primary care physicians’ knowledge of COPD and asthma around the world^22–27^, and our conclusions were consistent with them. Knowledge assessment studies of COPD have also been carried out in China, but in a relatively smaller sample size and were only regionally based^28,29^. Nevertheless, seldom studies focused on knowledge assessment of CAP and influenza either in the world or in China. There was one study which assessed the knowledge of CAP in China, but this was in a single city^30^.

Universal health coverage (UHC) is the cornerstone of good health and well-being for all, and it is underpinned by high-quality care, however, one of the biggest barriers to improved quality is the paucity of data on quality^31^. Training and upskilling community health workers, nurses, and physicians must remain a global priority^31^. All of this requires attention to developing active learning systems. But that does not just happen—it requires careful investment and design^32^. This study identified weaknesses in the knowledge of primary care physicians and provided precise directions for later training. An updated education program would be designed based on our findings. The model from China would be an example for other resource-limited countries.

This was the first and largest study to assess primary care physicians’ knowledge on CAP, asthma, influenza, and COPD simultaneously, with such a huge sample and broad geographical areas in China in the pre-epidemic era of COVID-19. Although only 65.1% (4815) of the questionnaires were analyzed finally, the sample size of the study was still the largest of its kind. Though the sample population was unevenly distributed, it was sufficient to get an overview of the current knowledge level of primary care physicians about these four common respiratory diseases.

The survey had some limitations. Firstly, as most of the academic activities were held in the north of China in the past two years, obviously, there was an over-representation of respondents from north China compared to south China (70.3% vs 0.3%). Thereby the generalizability of the findings for some provinces or cities was limited. Secondly, we did not have data on non-responders so we were unable to test for response bias. For a more comprehensive view to present a thorough picture of knowledge level of primary care physicians in specific areas, surveys like this but with more physicians to take part were needed so as to provide more individualized and targeted training for certain regions. Thirdly, the questionnaire revealed only theoretical knowledge and self-reported, preferred actions. Respondents may have reported what they believed to be acceptable, instead of what they actually practiced in their clinical work. What primary care physicians did in work settings remains unknown.

In conclusion, physicians in primary care in China had a poor knowledge of asthma, CAP, COPD, and influenza. This study suggested a clear need for further education for physicians in primary care on common respiratory diseases.

## Methods

We carried out the online questionnaire survey among primary care physicians in China. As this was an online study, participants did not provided written informed consent to take part in, but Informed Consented and Privacy Protection Terms were displayed on the head page of the e-questionnaire. Participants could go to answer questions if they ticked Consent.

### Measures

The questionnaire consisted of two parts, demographic characteristics information and questions about the knowledge of CAP, asthma, influenza, and COPD. Q1–Q10 were questions about demographic characteristics, including gender, age, education level, years of experience in practice, current vocational title, specialty, region, and grade of institutions. Knowledge survey questions were assessed using items adapted from the relevant guidelines^5,13,33,34^ and focused on four core aspects: (1) epidemiology and clinical characteristics/features; (2) diagnostic criteria; (3) treatment; (4) preventive measures from Q11 to Q47. Open questions were also used to explore their training needs for the four diseases, and the results would be reported in another paper in the near future. Specific questions were shown in supplementary file 1. An indefinite proportion of single-option and multi-option questions with 5–6 wrong answers per question were interspersed with each other. Each question had 5 points. For multi-option questions, respondents cannot get 5 points unless they picked out all the correct answers.

### Participants and procedure

As the aims of the study were descriptive, we did not carry out a formal sample size calculation but aimed to recruit as many participants as possible from a range of geographical locations. A QR-Code and web link of the questionnaire (online supplementary file 1) were created by Wenjuanxing, a research consultancy specializing in online research. The questionnaire was administered to attendees of conferences, training courses, Clinical Knowledge and Skill competition, and academic activities held by CARDPC across the country in 2017 and 2018. Before the above activities, attendees were fully mobilized to answer the questionnaire by scanning the QR-Code via the prominent social application Wechat or by clicking the web link on laptop or mobile phones. Participation was by choice of the individual. Attendees who agreed to take part in the survey were only able to complete the questionnaire once and then submitted on-line. On-site investigators were available to help participants to complete the questionnaire if necessary. All respondents were anonymized. Data collection continued from July, 2017 to December, 2018, the questionnaire was available at any time during this time period.

The questionnaire was developed by a group of respiratory physicians from CARDPC which convened respiratory experts from all over the country. 534 respiratory physicians from general hospitals and physicians from primary care were invited to pretest the questionnaire which resulted in some adjustments and changes to improve it. Finally, the questionnaire comprised 12 questions on COPD with a full score of 60 points, 9 questions on asthma with a full score of 45 points, 8 questions on CAP with a full score of 40 points, 9 questions on influenza with a full score of 45 points. The full score of the whole questionnaire was 190. If the total score of the four diseases exceeded 114 points (60% of 190 points), it was considered as “pass”, which meant that the respondent had basically mastered the knowledge of the four diseases. When respondents submitted the questionnaire, the total score and scores for each disease would be calculated automatically by and stored in the database of Wenjuanxing. In order to avoid the psychological impact on the respondents and in consideration of privacy protection issue, the scores were not displayed to respondents when they finished the submission.

### Analysis

The data were exported from the database of Wenjuanxing by a designated researcher. Descriptive statistics were used to present demographic characteristics of respondents. Continuous variables were expressed as the mean (±standard deviation), and categorical variables were presented as relative frequencies and percentages (*n*, %). Correct rates of individual questions were compared by community and township hospitals using chi-squared statistics. The *t*-test and 95% confidence intervals (CIs) were used to assess whether differences in scores between the two different medical facilities were significant. 𝑝 values <0.05 were considered as statistically significant. No imputation was performed for missing data.

Microsoft Excel and SPSS20.0 software were used for statistical analysis.

### Ethics approval

The study was approved by Peking University First Hospital Research Ethics Committee (REC) (2020-036).

### Reporting summary

Further information on research design is available in the Nature Research Reporting Summary linked to this article.

## Supplementary information

Supplementary information

REPORTING SUMMARY

## Data Availability

The data that support the findings of this study are available from the corresponding authors upon reasonable request.
