# Supplementary material for: An online survey of primary care physicians’ knowledge of common respiratory diseases in China
Source: NPJ Prim Care Respir Med. 2022 Aug 19;32:28. doi: 10.1038/s41533-022-00289-5 (PMC9389487; doi:10.1038/s41533-022-00289-5)
Supplement: Supplementary file 1 — Supplementary information [file 41533_2022_289_MOESM1_ESM.pdf]

Questionnaire of common respiratory disease knowledge

The questionnaire comprised 12 questions(questions 11 to 22) on COPD with a full score of 60 points, 9 questions(questions 23 to 31) on asthma with a full score of 45 points, 8 questions(questions 32 to 39) on CAP with a full score of 40 points, 9 questions(influenza) on influenza with a full score of 45 points, the total score of the whole questionnaire is 190 points. For multi-option questions, participants can get all the 5 points if they pick out all the correct answers.

Part I: Basic information

1. Institution address*
____

2. Name of your institution*: ______________________________

3. Institution grade: [single choice]*
○Level 1 ○Level 2 ○Level 3 ○Class A ○Qualified

4. Age: __________*

5. Gender: *
○Male ○Female

6. Education level:*
○Master
○Bachelor
○Junior College
○High school and below

7. Experience years: __________ years*

8. Vocational Title: *
○Senior title
○Intermediate title
○Junior title
○No title.

9. Specialty: *
○ General practitioner
○Internal Physician (not specialized)
○ Respiratory physician
○ Specialist (Western medicine, non-respiration)
○ TCM Traditional Chinese Medicine
○Public health physician
○Nurses
○ Other, please indicate__________________

10. The country you are from:______________

Part II: Chronic Obstructive Pulmonary Disease(COPD)

11. The characteristic symptoms of COPD are:*
A. chronic cough and expectoration (correct answer)
B. hemoptysis
C. chest pain
D. exertive dyspnea (shortness of breath, suffocation) (correct answer)
E. unclear

12. The most important environmental factors of COPD: [single choice]*
A smoking (correct answer)
B air pollution
C occupational dust and chemicals
D biofuel smoke
E infection
F unclear

13. In the diagnosis of COPD, if the FEV1 / FVC is lower than ______ after inhalation of bronchodilator, it can be determined as persistent airflow restriction: [single choice]*
A 80%
B 70% (correct answer)
C 50%
D 30%
E unclear

14. The gold standard for diagnosing COPD is: [single choice]*
A symptoms
B signs
C lung function (correct answer)
D chest X-ray or CT
E unclear

15. The standard of FEV1 in predicted value (%) of class II (moderate airflow restriction) COPD is: [single choice]*
A <30%
B 30~49%
C 50-79% (correct answer)
D > 80%
E unclear

16. The main treatment measures to control the symptoms of COPD are: [single choice]*
A expectorant
B hormone
C immunomodulator
D bronchodilator (correct answer)
E unclear

17. The main bronchodilators for the treatment of COPD include:*

A β2 receptor agonist (correct answer)
B anticholinergic (correct answer)
C methylxanthines (correct answer)
D phosphodiesterase 4 (PDE-4) inhibitor
E unclear

18. The daily duration of long-term family oxygen therapy is: [single choice]*
A >8 hours
B >10 hours
C > 15 hours (correct answer)
D 24 hours
E unclear

19. The management objectives of COPD in stable phase are:*
A to relieve symptoms (correct answer)
B to improve health condition(correct answer)
C prevention of disease progression (correct answer)
D prevention and treatment of acute aggravation and reduction of mortality (correct answer)
E unclear

20. Management of COPD in stable phase includes:*
A education and management (correct answer)
B control of occupational or environmental pollution (correct answer)
C medication (correct answer)
D oxygen therapy (correct answer)
E ventilation support (correct answer)
F rehabilitation treatment (correct answer)
G surgery (correct answer)
H unclear

21. The most common cause of acute exacerbation of COPD is: [single choice]*
A arrhythmia
B congestive heart failure
C pneumonia
D infection of trachea and bronchus (correct answer)
E unclear

22. The following non-pharmacological treatment measures for COPD are:*
A quitting smoking (correct answer)
B control of occupational or environmental pollution (correct answer)
C oxygen therapy (correct answer)
D noninvasive ventilation (correct answer)
E breathing exercise (correct answer)
F nutritional support (correct answer)
G unclear

Part three: bronchial asthma

23. The main clinical manifestations of asthma include recurrent:*

A puffing (correct answer)
B dizziness
C. shortness of breath (correct answer)
D chest tightness (correct answer)
E cough (correct answer)
F unclear

24. Which of the following conditions must be met simultaneously for the diagnosis of asthma:*
A repeated attacks of wheezing, shortness of breath, chest tightness, cough, etc,which are mostly related to contact with allergens, cold air, physical and chemical stimulation, upper respiratory tract infection, exercise, etc. (correct answer)
B. both lungs can hear scattered or diffuse wheezing sound mainly in expiratory phase (correct answer)
C. symptoms and signs can be relieved by treatment or by themselves (correct answer)
D unclear

25. The examination that can assist in the diagnosis of asthma includes:*
A allergen skin test
B peak current meter (correct answer)
C bronchial provocation test (correct answer)
D bronchodilation test (correct answer)
E measurement of pulmonary ventilation function (correct answer)
F unclear

26. The normal value of peak flow meter evaluation is: [single choice]*
A PEF accounted for more than 80% of predicted value or personal best value, and the variation rate was less than 20% (correct answer)
B PEF accounts for 60% ~ 80% of the predicted value or personal best value, and the variation rate is 20% ~ 30%
C PEF accounted for more than 60% of predicted value or personal best value, and the variation rate was less than 20%
D unclear

27. Among the drugs for asthma treatment, the first choice of control drugs is: [single choice]*
A inhaled corticosteroids (ICS) (correct answer)
B leukotriene regulator
C long acting $\beta 2$ receptor agonist
D slow release theophylline
E unclear

28. The remission drugs for asthma are:*
A fast inhaled $\beta 2$ receptor agonist (correct answer)
B systemic glucocorticoids (correct answer)
C inhaled short acting anticholinergic drugs (correct answer)
D theophylline (correct answer)
F unclear

29. The treatment options for patients with mild asthma who have not been treated in the past are as follows:*
A low dose inhaled corticosteroids, short acting $\beta 2$ receptor agonists on demand (correct answer)

B slow release theophylline, short acting β2 receptor agonist on demand (correct answer)
C minimum dose of glucocorticoid, slow-release theophylline, short acting β2 receptor agonist
D low dose inhaled glucocorticoid, LABA (aerosol), short acting drugs on demand
E unclear

30. The principles for the treatment of asthmatic attack are as follows:*
A correcting hypoxia (correct answer)
B timely and sufficient systemic use of glucocorticoids (correct answer)
C low dose inhaled corticosteroids
D antispasmodic and antiasthmatic (correct answer)
E remove incentives (correct answer)
F unclear

31. Prevention and management measures of asthma include:*
A avoiding or reducing exposure to risk factors such as indoor and outdoor allergens, virus infection, pollutants, tobacco smoke, drugs, etc. (correct answer)
B. carrying out asthma knowledge education to improve self-care awareness and ability of asthma patients (correct answer)
C. developing individualized treatment plan, self-monitoring and evaluation of patients (correct answer)
D long term follow-up management
E unclear

Part four: community-acquired pneumonia (CAP)

32. The clinical diagnosis basis of community-acquired pneumonia is as follows:*
A cough (correct answer)
B expectoration (correct answer)
C hemoptysis
D dyspnea
E fever (correct answer)
F signs of pulmonary consolidation (correct answer)
G wet rales (correct answer)
H X-ray examination of the chest shows infiltrative shadow of flake and patchy or interstitial change (correct answer)
I unclear

33. Etiological diagnosis specimens include:*
A urine (correct answer)
B feces
C phlegm (correct answer)
D blood (correct answer)
E lung biopsy specimen (correct answer)
F serum (correct answer)
G unclear

34. What are the conditions that CAPpatients need etiological examination*
A mild CAP
B clustering disease (correct answer)

C initial empirical treatment is invalid (correct answer)
D inpatient CAP patients (correct answer)
E with pleural effusion (correct answer)
F unclear

35. The diagnostic criterion of severe pneumonia are as follows:*
A needing tracheal intubation for mechanical ventilation (correct answer)
B septic shock still needs vasoactive drug treatment after active fluid resuscitation (correct answer)
C respiratory rate ≥ 30 times / min; PaO2 / FiO2 ≤ 250mmHg (1mmhg = 0.133kpa); multi pulmonary lobe infiltration (correct answer)
D urea nitrogen > 7 mmol / L; respiratory rate ≥ 30 times / min; systolic blood pressure < 90 mmHg requires active fluid resuscitation (correct answer)
E unclear

36. The common pathogens of CAP are:*
A Streptococcus pneumoniae (correct answer)
B Haemophilus influenzae (correct answer)
C Mycoplasma pneumoniae (correct answer)
D Legionella pneumophila (correct answer)
E Staphylococcus (correct answer)
F unclear

37. The first empiric anti infective drugs for young and middle-aged patients without basic diseases are:*
A aminopenicillin, penicillin / enzyme inhibitor complex (correct answer)
B first and second generation cephalosporins (correct answer)
C tetracyclines (correct answer)
D penicillin / enzyme inhibitor complex, second-generation cephalosporins, third-generation cephalosporins combined with tetracyclines / macrolides
E breathing quinolones (correct answer)
F macrolides (correct answer)
G unclear

38. The patients' condition and diagnosis should be evaluated 48-72 hours after the initial treatment of community-acquired pneumonia, the response to treatment are as follows: *
A hypothermia (correct answer)
B. improvement of respiratory symptoms (correct answer)
C leukopenia (correct answer)
D Absorption of lesions on X-ray chest radiograph (correct answer)
E systolic pressure ≥ 90mmHg
F unclear

39. What are the preventions of pneumonia*
A quitting smoking (correct answer)
B avoiding drinking (correct answer)
C vaccinating streptococcus pneumoniae vaccine (correct answer)
D vaccination against influenza (correct answer)
E weight control
F unclear
Part V: influenza

40. Symptoms and signs of simplex influenza are as follows:*
A sudden onset (correct answer)
B high fever (correct answer)
C diarrhea
D vomiting
E chills (correct answer)
F pharyngeal pain (correct answer)
G muscle soreness (correct answer)
H unclear

41. The epidemiological characteristics of influenza are as follows:*
A sudden outbreak (correct answer)
B no seasonality
C spreads rapidly (correct answer)
D high incidence (correct answer)
E low mortality (correct answer)
F influenza A often appears in the form of epidemic (correct answer)
G unclear

42. The main infection routes of influenza are:*
A air droplets (correct answer)
B direct or indirect contact with mucous membrane in mouth, nose and eyes (correct answer)
C contact with respiratory secretions, body fluids and articles contaminated with virus of patients (correct answer)
D drinking water
E insect vectors
F unclear

43. The diagnostic criterion for influenza are:*
A positive influenza virus nucleic acid test (correct answer)
B if the rapid antigen test of influenza virus is positive, a comprehensive judgment should be made in combination with the epidemiological history (correct answer)
C positive influenza virus isolation and culture (correct answer)
D the level of influenza virus specific IgG antibody in both acute and convalescent sera was three or more times higher
E unclear

44. The criterion of severe influenza are as follows:*
A change of mind (correct answer)
B. severe vomiting, diarrhea and dehydration (correct answer)
C dyspnea and / or increased respiratory rate (correct answer)
D polyuria
E blood pressure < 80 / 60mmHg
F arterial partial pressure of oxygen (PaO_2) < 60 mmHg or oxygenation index (PaO_2 / FiO_2) < 300 (correct answer)
G. the original basic diseases are obviously aggravated, with organ dysfunction or failure (correct answer)
H chest radiograph shows infiltration shadow of bilateral or multiple lobes, or the expansion by 50% or more of infiltration shadow of lung within 72 hours after admission

I the level of creatine kinase and its isoenzyme increased rapidly (correct answer)
J unclear

45. The most common and serious complications of influenza are: [single choice]*
A severe pneumonia (correct answer)
B Reye syndrome
C nervous system injury
D heart damage
E septicemia
F meningitis
G unclear

46. The most basic and important link of the treatment of influenza is: [single choice]*
A symptomatic treatment
B antiviral therapy (correct answer)
C antibacterial therapy
D unclear

47. What drugs can be used to treat influenza B:*
A zanamivir (correct answer)
B rimantadine
C oseltamivir (correct answer)
D amantadine
E unclear

48. The most effective way to prevent influenza and its complications is: [single choice]*
A strengthening personal hygiene
B flu vaccination (correct answer)
C antiviral therapy
D strengthening the prevention and control of influenza outbreaks in institutions
E maintaining indoor air circulation
F unclear
